# Dried bovine placenta improves spermatozoa count in a rat model of male reproductive aging

**DOI:** 10.14202/vetworld.2021.1602-1607

**Published:** 2021-06-21

**Authors:** Surya Agus Prihatno, Yosua Kristian Adi, Teguh Budipitojo, Topas Wicaksono Priyo Jr, Yonathan Alvin Maruli Asi Sihotang

**Affiliations:** 1 Department of Reproduction and Obstetric, Faculty of Veterinary Medicine, Universitas Gadjah Mada, Yogyakarta 55281, Indonesia; 2 Department of Anatomy, Faculty of Veterinary Medicine, Universitas Gadjah Mada, Yogyakarta 55281, Indonesia; 3 Sains Veteriner Magister, Faculty of Veterinary Medicine, Universitas Gadjah Mada, Yogyakarta 55281, Indonesia

**Keywords:** aging, bovine placenta, male reproductive, sperm count, testicular

## Abstract

**Background and Aim:**

In the male reproductive system, the aging process can lead to infertility. Recently, placenta and its derivatives have been researched as regenerative agents. This study aimed to describe the basic components of dried bovine placenta powder and its potential effects as a regenerative agent in a rat model of male reproductive aging with D-galactose induction.

**Materials and Methods:**

We divided 15 male Wistar rats, 2 months of age, into three groups: A, the health control group; B, the D-galactose induction group, and C, the D-galactose induction and 10% dried bovine placenta supplementation group. We measured epididymal sperm concentration and testicular weight and volume and analyzed these using one-way analysis of variance.

**Results:**

Dried bovine placenta was rich in nutrients, with 61.98% protein, 21.25±2.07 carbohydrates, 8.58% water, 4.93% ash, and 3.27% fat. The mean epididymal spermatozoa concentration of the rats in Groups A, B, and C was 3026×10^6^/mL, 1492.8×10^6^/mL, and 2732.5×10^6^/mL, respectively. The average total testicle weights were 2.44 g, 2.72 g, and 2.57 g, respectively. The average total testicle volumes were 2.29 cm^3^, 2.49 cm^3^, and 2.33cm^3^, respectively.

**Conclusion:**

Dried bovine placenta powder is rich in nutrients, especially protein. Supplementation with dried bovine placenta can improve epididymal spermatozoa concentration that is important in fertility.

## Introduction

Aging is a complex biological process involving organic, cellular, and molecular changes. This process is caused chronologically by increasing age but can also be accelerated by environmental factors that cause oxidative stress [1,2]. In the male ­reproductive system, the aging process can lead to infertility. Semen volume and sperm motility and concentration decline as age increases [3]. A decrease in the quality of bull semen due to aging is a problem at artificial insemination centers in Indonesia [4]. Older bulls often are retained because the cost to replace them is high. Supplementation to improve bull reproductive performance is an option, but it has not been done because limited research exists on regenerative agents for male livestock reproductive degeneration caused by aging.

Recently, placenta and its derivatives have been studied as regenerative therapy for various degenerative diseases. Placental components can include cord blood cells [5], mesenchymal stem cells [6,7], amniotic membrane [8], placental extract [9], cord blood serum [10], and amniotic fluid [11]. Dried placenta is also rich in protein, fiber, fat, minerals (e.g., sodium, potassium, phosphorus, calcium, iron, magnesium, zinc, copper, and manganese), and hormones (e.g., estradiol, progesterone, testosterone, and growth hormone) [12]. In traditional Chinese medicine, dried human placenta is one ingredient used to treat infertility in humans [13]. Compared to human placenta, bovine placenta is easier to obtain in large quantities. However, dried bovine placenta has never been studied for its nutritional content or its potential effects as a regenerative agent in infertility caused by aging.

Therefore, in this study, we describe the basic components of dried bovine placenta of beef cattle from a traditional farm in Yogyakarta, Indonesia. We analyzed the placenta for its potential effects as a regenerative agent in a rat model of male reproductive degeneration caused by aging. We used D-galactose as an inducting agent for male reproductive aging, adapting a method previously described by Salman *et al*. [14].

This study aimed to describe the basic components of dried bovine placenta powder and its potential effects as a regenerative agent in a rat model of male reproductive aging with D-galactose induction.

## Materials and Methods

### Ethical approval

The study was approved by the Research Ethics Committee, Faculty of Veterinary Medicine, Universitas Gadjah Mada with Ethical Clearance Letter Number: 0023/ EC-FKH/Int./2020.

### Study period and location

This study was conducted from June to November 2020 at Practical Animal Room and Laboratory of Reproduction and Obstetrics, Faculty of Veterinary Medicine, Universitas Gadjah Mada, Yogyakarta Indonesia.

### Dried bovine placenta

Fresh bovine placenta that was expulsed *<*6 h post-parturition was obtained from a traditional farm in Yogyakarta, Indonesia. The placenta was washed using sterile water, cut into a smaller piece, and dried under the sun or an oven until the water content decreased. Then, the placenta was ground into a powder.

Ten grams of bovine placenta powder were analyzed for water, ash, fat, protein, and carbohydrate content at the Universitas Gadjah Mada Center for Biotechnology Studies. For use as a supplementation, the placenta powder was mixed with A.D.II pellets in a ratio of 1:9 (10% bovine placenta in A.D.II pellets).

### Animal experiment

In this study, 15 male Wistar rats, aged 2 months, were used. The rats were adapted for a new environment from a breeding place to a practical animal room for 1 week and were maintained in a plastic box with fine air circulation in the room with a 12 h dark-light cycle. The room temperature ranged from 24.3°C to 33.3°C, with the lowest humidity at 39% and the highest at 87%. The rats were fed commercial A.D.II pellets and could drink fresh water *ad libitum*.

After acclimation, the rats were divided into three groups: Group A, healthy controls; Group B, receiving D-galactose induction; and Group C, receiving D-galactose induction with dried bovine placenta supplementation. The rats in Groups B and C were inducted with 3 mg/kg body weight D-galactose orally for 6 weeks. After this induction, the rats in Group C were supplemented with 10% dried bovine placenta mixed in the feed for 30 days. After the treatment, all the rats in all the groups were anesthetized to collect the required samples.

### Spermatozoa count

The testicular organ, along with the epididymis, was removed from the scrotum. The epididymis was separated from the testicular organ. As much as, 10 μL of semen from the cauda epididymis was taken using a micropipette. Semen was mixed with 990 μL of NaCl solution in a 1.5 mL tube. The semen solution was diluted again using a leukocyte pipette 101. Spermatozoa counting was done using a Neubauer chamber (Hemocytometer: ASSISTANT^®^, Germany). The calculation was carried out 3 times, and the results were averaged.

### Testes weight and volume

The removed testes were weighed using a digital scale (model: EHA401, CAMRY^®^, Zhongshan, Guangdong, China). After that, the testes were fixed in Bouin’s solution for 24 h. For preservation, the testes were moved in 70% alcohol solution. The length (l), height (h), and width (w) of the testes were measured using a Vernier caliper (Tricle Brand^®^, Shanghai, China). Testes volume (V) was measured using the following formula: 
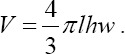
.

### Statistical analysis

We analyzed the bovine placenta contents descriptively and the spermatozoa count and testes weight and volume statistically using one-way analysis of variance. P values considered significant at p<0.05.

## Results

### Dried bovine placenta contents

The dried bovine placenta powder was rich in nutrients. The highest nutritional content was protein, with an average percentage of 61.98%. Carbohydrates were the second highest nutrient content at 21.25% average content. Water, ash, and fat were the lowest content, with 8.58%, 4.93%, and 3.27% average content, respectively (Table-1).

**Table-1:** Contents nutrient analysis of dried bovine placenta powder.

Samples	Content of dried bovine placenta powder				

	Water (%)	Ash (%)	Fat (%)	Protein (%)	Carbohydrates (by different %)
Bovine placenta powder 1	8.21 (8.25)	5.57 (5.86)	3.32 (2.79)	62.94 (63.84)	19.96 (19.26)
Bovine placenta powder 2	9.05 (8.79)	4.14 (4.16)	3.43 (3.53)	61.49 (59.65)	21.89 (23.87)
Average	8.58±0.41	4.93±0.91	3.27±0.33	61.98±1.83	21.25±2.07

### Spermatozoa count

The concentration of spermatozoa from the caudal epididymis was determined using a Neubauer chamber. The average spermatozoa count in the rats in Groups A, B, and C was 3026×10^6^/mL, 1492.8×10^6^/mL, and 2732.5×10^6^/mL, respectively. Group A rats had the highest spermatozoa concentration; Group B had the lowest (Table-2).

**Table-2:** The number of spermatozoa from the caudal epididymis.

Rat number	Spermatozoa count (10^6^/mL)		

	Group A	Group B	Group C
Rat 1	3466	1266	2266
Rat 2	3200	1200	3266
Rat 3	2600	1532	2666
Rat 4	2932	2000	-
Rat 5	2932	1466	2732
Average	3026±25	*1492.8±315	2732.5±411

* p<0.05

### Testes weight and volume

The weight of the left and right testicle was measured using a digital scale soon after removing them from the scrotal sac. The average total testicle weight of the rats in Groups A, B, and C was 2.44 g, 2.72 g, and 2.57 g, respectively (Table-3). The average total testicle volume of the rats in Groups A, B, and C was 2.29 cm^3^, 2.49 cm^3^, and 2.33 cm^3^, respectively (Table-4).

**Table-3:** Testicle weight of rats in Group A, Group B, and Group C.

Rat number	Testicle weight (g)					

	Group A		Group B		Group C	
		
	Left testis	Right testis	Left testis	Right testis	Left testis	Right testis
Rat 1	1.20	1.28	1.40	1.40	1.46	1.40
Rat 2	1.36	1.42	1.50	1.46	1.34	1.34
Rat 3	0.98	1.06	1.24	1.24	1.22	1.22
Rat 4	1.10	1.04	1.40	1.38	-	-
Rat 5	1.40	1.36	1.30	1.28	1.18	1.10
Average	1.21± 0.18	1.23± 0.17	1.37± 0.10	1.35± 0.09	1.30± 0.13	1.27± 0.13
Total	2.44±0.31		2.72±0.17		2.57±0.22	

**Table-4:** Testicle volume of rats in Group A, Group B, and Group C.

Rat number	Testicle volume (cm^3^)					

	Group A		Group B		Group C	
		
	Left testis	Right testis	Left testis	Right testis	Left testis	Right testis
Rat 1	1.25	1.08	1.26	1.31	1.28	1.35
Rat 2	1.30	1.24	1.33	1.31	1.18	1.11
Rat 3	1.00	1.01	1.17	1.13	1.12	1.14
Rat 4	1.00	1.03	1.24	1.29	-	-
Rat 5	1.26	1.26	1.23	1.19	1.09	1.04
Average	1.16± 0.15	1.12± 0.12	1.25± 0.06	1.25± 0.08	1.17± 0.08	1.16± 0.13
Total	2.29±0.23		2.49±0.12		2.33±0.18	

## Discussion

In mammals, a placenta is an organ for the maternal-fetal exchange of gas and nutrients, hormone secretion, and immune responses [15]. The gross anatomic structure of the bovine placenta is classified as the cotyledonary type [16]. The bovine placenta has 75-125 placentomes. Histological classification of the bovine placenta in early pregnancy has been known to be the epitheliochorial type, which becomes progressively synepitheliochorial at the beginning of the second trimester [17]. In this study, we collected placenta that had been expelled <6 h earlier. The bovine fetal membrane is normally expelled within 12 h after delivery of the calf.

Analysis of the dried bovine placenta powder showed that it was still rich in nutrients. This finding is in line with a report by Phuapradit *et al*. [12] on the nutrients and hormones in a heat dried human placenta, which was rich in protein and minerals. However, the protein hormone concentration was reduced during the steaming and dehydration processes [18]. At least 16 hormones can be detected in human placenta samples processed for encapsulation, and some in concentration can induce physiological effects [19]. In addition, antioxidant peptides can be extracted from human and goat placenta [20,21]. In this study, 10% dried bovine placenta in commercial feed could increase the protein content from 15.39% to 22.95%.

**Figure-1:**
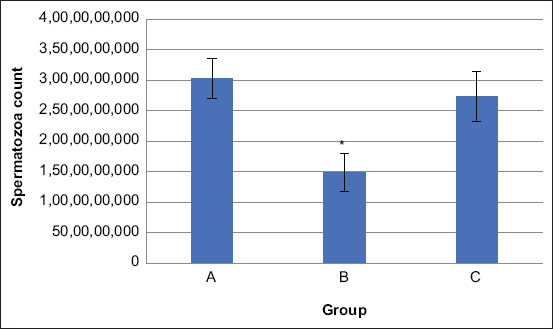
Concentration of spermatozoa from cauda epididymal semen of rats in Group A, Group B, and Group C. Statistical analysis showed significant difference (*) with p<0.05.

Our statistical analysis of the spermatozoa count from the epididymal semen showed a significant difference among the groups. Spermatozoa concentration in Groups A and C was significantly different from that in Group B (p<0.05). However, we found no significant difference in the spermatozoa concentration of the rats in Groups A and C (Figure-1). This result confirmed an earlier finding that the administration of D-galactose at a dosage of 3 mg/kg Body Weight orally for 6 weeks can reduce epididymal sperm count [14]. The administration of D-galactose systemically can induce aging *in vitro* and *in vivo* and has been widely utilized in anti-aging therapeutic studies [22].

Many researchers have studied the role of ­oxidative stress in D-galactose-induced animal models [23-29]. In our study, we proved that supplementation with dried bovine placenta can improve spermatozoa count in rats induced into aging by D-galactose. However, the mechanisms involved in the improvement of spermatogenesis are still unclear. During pregnancy, the placenta produces cytokines such as tumor necrosis factor (TNF)-alpha, interleukin (IL)-1, IL-6, and IL-10 [30-32]. Cytokines, which are small proteins secreted and released by cells, have a specific effect on the interaction and communication between cells [33]. Cytokines also have an important role in spermatogenesis.

TNF produces Sertoli cells (Scs) and germ cells within the testis to stimulate and maintain spermatogenesis [34]. IL-1 secreted by Scs and germ cells is known to inhibit Leydig cell steroidogenesis, stimulate Sc transferrin and IL-6 production, and promote Sc proliferation [35]. IL-1 along with IL-6 can regulate Sc and spermatogenic cell development [36]. In addition, IL-10 has been proposed to maintain an immune tolerant testicular environment due to its immunosuppressive properties [37].

The placenta produces large amounts of estrogen during human and non-human primate pregnancy, especially in late gestation [38]. In males, estradiol also is essential for modulating libido, erectile function, and spermatogenesis, although inappropriate estradiol levels can lead to a decrease in testicular size and sperm production [39]. However, cytokines and hormone concentrations in dried bovine placenta need to be investigated further.

**Figure-2:**
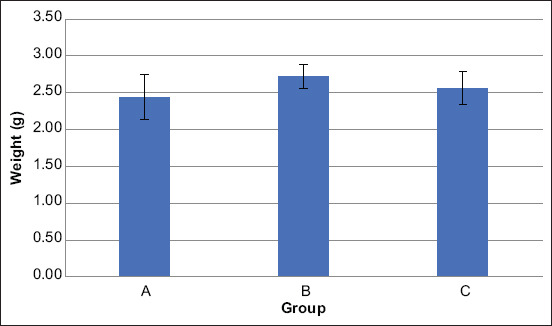
Average of testicle weight of rats in Group A, Group B, and Group C. Statistical analysis showed no significant difference.

**Figure-3:**
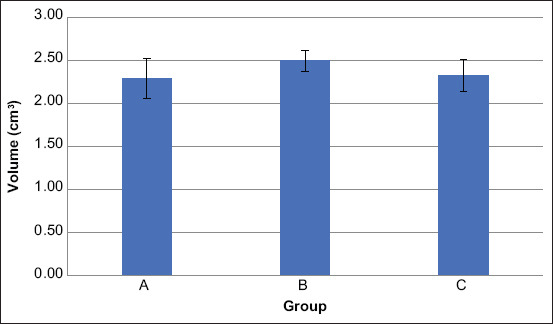
Average of testicle volume of rats in Group A, Group B, and Group C. Statistical analysis showed no significant difference.

Statistical analysis of testicle weight and volume of rats in Groups A, B, and C showed no significant differences, although the average sperm concentration from the cauda epididymal among the groups was significantly different. The average testicle weight and volume of the rats in Group B were slightly higher than those (Figures-2 and 3) of the rats in Groups A and C. Similar results have been previously reported by Amini *et al*. [40], indicating that no significant differences were found in the testicular weight and volume of rats fed a sesame seed diet although the mean cell number in the left epididymis was significantly different. Trautwein *et al*. [41] reported that testicle volume and weight did not influence the quality of the epididymal sperm of cats. Other reports have stated that some factors such as age, season, environment, and body size can influence testicular weight and volume [42-44]. In our study, we controlled for age, season, and environment.

## Conclusion

Bovine placenta is rich in nutrients, especially protein. Supplementation of 10% dried bovine placenta powder in a rat model of male reproductive aging induced by D-galactose can improve epididymal spermatozoa concentration that is important in fertility.

## Authors’ Contributions

SAP and YKA: Designed the experimental procedures. TWPJ and YAMAS: Conducted the research work. YKA and TB: Performed sample and statistical analysis. SAP, YKA, and TB: Drafted the manuscript. All authors read and approved the final manuscript.
